# Strategies to prevent HIV transmission among heterosexual African-American men

**DOI:** 10.1186/1471-2458-5-3

**Published:** 2005-01-07

**Authors:** Ekere J Essien, Angela F Meshack, Ronald J Peters, Gbadebo O Ogungbade, Nora I Osemene

**Affiliations:** 1 The HIV Prevention Research Group. College of Pharmacy, University of Houston, 1441 Moursund Street, Houston, Texas 77030, USA; 2 Center for Health Promotion and Prevention Research. University of Texas School of Public Health, Houston, Texas 77030, USA; 3 College of Pharmacy, Texas Southern University, Houston. Texas 77004, USA

**Keywords:** African Americans, men, HIV, AIDS, risk behaviors

## Abstract

**Background:**

As part of qualitative research for developing a culturally sensitive and developmentally appropriate videotape-based HIV prevention intervention for heterosexual African- American men, six focus groups were conducted with thirty African-American men to determine their perceptions of AIDS as a threat to the African-American community, characteristics of past situations that have placed African Americans at risk for HIV infection, their personal high risk behaviors, and suggestions on how HIV intervention videotapes could be produced to achieve maximum levels of interest among African-American men in HIV training programs.

**Methods:**

The groups took place at a low-income housing project in Houston, Texas, a major epicenter for HIV/AIDS. Each group was audiotaped, transcribed, and analyzed using theme and domain analysis.

**Results:**

The results revealed that low-income African-American men perceive HIV/AIDS as a threat to their community and they have placed themselves at risk of HIV infection based on unsafe sex practices, substance abuse, and lack of knowledge. They also cite lack of income to purchase condoms as a barrier to safe sex practice. They believe that HIV training programs should address these risk factors and that videotapes developed for prevention should offer a sensationalized look at the effects of HIV/AIDS on affected persons. They further believe that programs should be held in African-American communities and should include condoms to facilitate reduction of risk behaviors.

**Conclusions:**

The results indicate that the respondents taking part in this study believe that HIV and AIDS are continued threats to the African-American community because of sexual risk taking behavior, that is, failure to use condoms. Further, African-American men are having sex without condoms when having sex with women often when they are under the influence of alcohol or other mind-altering substances and they are having sex with men while incarcerated and become infected and once released resume unprotected sexual relations with women. According to the men, substance abuse is an important part of the problem of HIV in the African-American community. This is in keeping with research that shows that drug use, especially crack cocaine, is linked to sexual risk taking among African Americans and to increased likelihood of becoming infected with other sexually transmitted diseases (STDs) including HIV. Thus, interventions for men should address condom use, condom availability, skills for using condoms, eroticizing condoms and substance abuse prevention. Men in the present study also strongly recommended that HIV/AIDS videotaped messages should include footage of the sensational effects of the disease.

## Background

The HIV/AIDS epidemic continues to be a major public health challenge in the African-American community. Although African Americans constitute only 13% of the United States population, they have accounted for 39% of all the 886,000 estimated AIDS cases that have been diagnosed since the epidemic began in 1981 [1]. In 2002, African Americans comprised about 50% of the 42,000 AIDS cases diagnosed among adults in the United States, and also accounted for more than half of the HIV diagnoses that were reported to the Centers for Disease Control and Prevention (CDC) [1]. Amongst these, the rate of diagnoses among African-American men was almost nine times greater than the rate for white men [1]. The CDC has identified sexual contact between African-American men who have sex with men, injection drug use, and heterosexual contact respectively, as the leading causes of HIV infection among this group [1].

While several successful intervention programs have been developed, evaluated, and implemented for African-American men who have sex with men, African-American women, and African-American adolescents [2], only a limited number of prevention programs have been implemented for heterosexual African-American men [2]. This is unusual given that heterosexual transmission has been identified as the leading cause of HIV infection among African-American women [1].

Several factors other than those reported by the CDC have placed African-American men at an increased risk of HIV infection. Essien et al. [3] explored the role of misperceptions about HIV transmission among African-American men and women by conducting eleven focus groups with sixty-nine men and women. They reported myths and misperceptions about HIV transmission such as denial of personal risk, perceptions that HIV was a disease that happened to outsiders and "others" and the perceived role of the government in the development of HIV as contributing factors to the efficiency of HIV transmission in this population. In a follow-up study to assess the range of discrepancy in self-reported sexual identity and sexual behavior in men and women of four racial/ethnic groups, Ross et al. [4] found that 43% of the African-American men in their sample who self-identified as heterosexuals were also having sex with other men. This finding is consistent with a recent report by Montgomery et al. [5] which showed that, among African-American men who had sex with men, 34% had also been engaged in a bisexual relationship. Research has shown that bisexual African-American men may not relate to HIV prevention messages that have been developed for gay men [1]. Thus, intervention programs for African-American men must be innovative and firmly grounded in the socio-cultural contexts of sexual risk taking in this population.

An array of socioeconomic and cultural factors exacerbates high-risk behaviors that place African-American men at risk for HIV. First, nearly 25% of African Americans live in poverty [6], and several studies have shown a direct relationship between poverty and increased prevalence of HIV/AIDS [7-10]. This may be due to high rates of unemployment, associated drug related economic activities, money-for-sex and drugs-for-sex exchanges, and inadequate access to health care and HIV prevention programs [1,11,12]. Secondly, injection drug use has been identified as the next leading cause of HIV infection among African-American men [1]. Aside from sharing injection paraphernalia, African Americans who are substance abusers often engage in high risk behaviors such as unprotected sex when they are under the influence of drugs and alcohol [13]. Thirdly, cultural and social norms in African-American communities are not supportive of homosexual behavior. It has been argued that negative attitudes associated with homophobia may lead to psychological distress and sexual risk taking among African-American men leading them to having relationships with both women and men [14].

A major challenge facing HIV prevention interventionists is the issue of developing HIV prevention programs that are firmly grounded in the cultural nuances of African Americans and the translation of these programs into effective HIV prevention practice. To date, several HIV intervention programs have been developed for African Americans [15-18], and the most promising interventions have been programs that are based on social cognitive principles. However, Kalichman et al. [19] have noted that HIV prevention programs that are based on social cognitive principles and proven to be effective in the scientific literature have not been widely utilized because of their reliance on expert interventionists for implementation in face-to-face formats, making them difficult to transfer to community-based organizations [19]. In contrast, social cognitive theory principles applied to HIV prevention can be delivered effectively by videotape and community-based organization personnel with minimal training in skills building techniques [19]. The rationale for using videotapes as part of an HIV intervention delivery system is provided by the emerging literature which demonstrates the feasibility of this medium in changing high risk sexual behaviors [20-23]. The research presented here results from qualitative studies conducted in Houston, Texas, to examine the sociocultural contexts of sexual risk taking among African-American men and to determine how a videotape-based HIV prevention intervention could be tailored so that it is effective in preventing HIV transmission among African-American men.

## Methods

### Design

This study used a qualitative exploratory research design to elicit information about the strategies for preventing HIV transmission among African-American men, ages 18–29. Thirty low-income African-American men participated in five focus groups that were conducted at a housing project targeted for convenience sampling and because of its location in close proximity to the research institution in Houston, Texas, a leading HIV/AIDS epicenter in the United States [1]. Approval for the study was obtained from the relevant university Committee for the Protection of Human Subjects.

### Procedure

The investigative team recruited focus group participants by displaying posters and flyers at strategic locations in the housing project identified by the manager. The flyer listed the study inclusion criteria: African-American heterosexual male, aged 18 – 29, self-reported unprotected vaginal sex in the last six months or having been diagnosed or treated for a sexually transmitted disease in the past year. The flyer also listed a university telephone number that prospective participants could call to obtain additional information and/or schedule their participation in a group. When calls were received, a trained research assistant confirmed eligibility. Those selected for participation were then encouraged to invite their friends to participate. Of the 97 prospective participants who contacted the university, 54 agreed to participate. Of that number, 30 appeared on the scheduled day and formed the study sample. The focus groups were conducted at the housing project's clubhouse by a facilitator with approximately 15 years experience conducting qualitative research studies with African-American men. Assistance was provided by a trained research assistant who took notes to ensure that pertinent information not captured by the audiotape was obtained and to record major points discussed.

Participants were informed of the benefits of their involvement, that involvement was voluntary, that they could refuse to answer any question, and that they could cease participation at any time without penalty. Prior to the start of each group, written informed consent was obtained and agreement obtained to record the session. The participants were also told about the confidentiality of the information discussed at the meeting, especially important given that some of the men were acquaintances as a result of residency in or near the housing complex. They were advised that they would receive a $25 mall gift certificate and condoms as incentives for their participation. Respondents were also advised that the tapes, which were anonymous, would be destroyed following transcription and checking. All questions as well as the informed consent were provided in English.

### Data collection

The investigative team utilized semi-structured and open-ended questions to elicit information from participants based on our research interest in determining perceptions of AIDS as a threat to African-American communities, characteristics of past situations which placed African Americans at risk for HIV infection, suggestions on ways to recruit African Americans into HIV/AIDS prevention programs and on how HIV intervention videotapes could be produced to achieve maximum levels of interest.

A three-step staging process was used to generate the questions used in the focus groups: (1) generation of working hypotheses on facilitators and barriers to HIV prevention and program messages and methods by conducting interviews with six key informants experienced in HIV/AIDS prevention among African Americans; (2) reshaping of hypotheses until no new information was received by testing them in interviews with men similar to the target population; and (3) using the resulting hypotheses to generate questions and field testing them with members of the target population after which final changes were made.

A guide consisting of a written list of questions and probes was used to conduct the interviews. According to McCracken [24], the advantages of using such a guide include the assurance that all areas of interest are covered and allowing the researcher to focus his attention on listening to the informants, thereby enabling a better understanding of their lines of thought and possibly unanticipated explanations of the concepts. The duration of each group was about two hours.

### Data analysis

The standard grounded theory approach of Glaser and Strauss was performed to conduct data analysis [25]. Grounded theory procedure and techniques were utilized for data analysis by doing a line-by-line analysis of the focus group transcripts and identifying the emerging themes [26]. The thematic concepts representing ideas expressed by a majority of the members of three or more focus groups were characterized as a domain and are reported below.

## Results

### Study participants

The study sample was comprised of 30 men between the ages of 18 and 29. Educational attainment ranged from tenth grade to college graduate with the greatest number of men stating that they had at least one year of post-secondary education. Most men identified themselves as day laborers attempting to earn income by doing various odd jobs as they become available. The remainder was unemployed. No men identified themselves as HIV positive, and none of the respondents had health insurance.

### Perception regarding AIDS and African Americans

The situational determinants of HIV risk taking and their impact on HIV/AIIDS prevention behaviors and education programs were examined. The discussion began by asking the men for their overall view regarding HIV and its impact on the African-American community. Specifically, they were asked if they believe that AIDS is a threat to the African-American community and to what they attribute its rapid spread. About one-half of men stated that it is due to lack of unsafe sex practices including having sex with multiple partners and failure to use condoms. A large number of men believed that African Americans have higher rates of HIV and AIDS due to their use of alcohol and drugs while a smaller number stated that AIDS is not a threat to the African-American community, but rather it is a universal problem or that African Americans have feelings of invincibility because they think that HIV happens to others and that God will protect them. A lesser number attribute the rapid spread of HIV and AIDS among African Americans to incarcerated men having sex with men while behind bars and returning to their female partners when they are released.

Clarence, a 26-year old forklift operator, believes that AIDS is a threat to the African-American community due to failure to use protection from lack of understanding about the disease:

"*A lot don't understand the disease or what the effect of it may be so they have unprotected sex because they don't understand the disease really well as they supposed to.*"

Ben, a 22 year old promotional worker, expressed his concerns about men having sex with men and also with women. He states:

"*It is not a problem on the outside, but a lot of men have sex without condoms in the jails and they are bringing it right back home, and they are not telling anybody that they had sex in jail, and they are bringing it and sharing it among the girls.*"

Kenny, a 27 year old temporary worker, adds:

"*I was recently locked up a couple of years ago. I met some guys you'd never think would be gay, undercover homosexuals, they pump weights with you, run track, and play basketball with you, but as soon as they are behind closed doors, their cellmate might be gay and they are banging in there. Even black gangs, sometimes when they initiate them, they do that, and they bring the disease back home.*"

Thus, incarceration and gang membership may facilitate HIV transmission and may be partially responsible for the high rates of bisexual activities in the African-American community.

Jason, a 20 year old laborer explained that AIDS is a universal threat but that it is an epidemic among African Americans due to associated substance abuse and failure to use protection:

"*It's a threat to everyone but we have a tendency in the black race, especially now with another epidemic that we have in our neighborhood called crack. People have a tendency now to be even careless with this drug. So they do things that they would not naturally do. Just having sex. The trust thing – we feel that we won't use condoms because we feel like they're uncomfortable, yeah, they kill the sensation and so then we're gonna jump in and do the do and so it has a stronger effect on us now than a lot of races.*"

Terry, a 28-year old home improver, asserts that African Americans do not believe that God will inflict added suffering (e.g., AIDS) upon them because they have already experienced hardship, God would not cause something else to make life difficult. He said:

"*It's because of the color of our skin. People believe that, seems to believe that, oh, God is going to let them know or it's not going to happen to me or something like this. We done had enough bad happen to us.*"

Karl, a 29 year old college graduate did not believe that AIDS is a threat to the African-American community, but rather that statistics are inflated for this group due to the places where they obtain medical treatment. He believes the government keeps data that private physicians do not which results in inflated numbers for African Americans. Mistrust of healthcare system reporting about African Americans leads him to believe the situation is not as bleak as reports indicate. He stated:

"*Most African Americans go to clinics which are state or federally funded and they keep that data. And most people with private health insurance go to their doctors and those private doctors don't submit that same data. So when you look at the quote-unquote per capita, it always looks like it is higher in the African-American community.*"

### Risk situations for HIV transmission

When asked to describe the means by which they had personally placed themselves at risk for HIV infection, almost half of participants cited unprotected sex or not taking the time to use protection "in the heat of the moment." These men did not have condoms in their possession, did not take the time to use a condom, or simply disregarded condom use because of dislike for them. Similarly, about one-third of men described specific situations with risks known to them at the time they engaged in unprotected intercourse. They included having sex with an HIV infected person, having sex with a stranger, sex with a drug user, and sex in exchange for money or drugs. The remainder of men stated that their drinking and drug use had created HIV risk situations.

Austin, a 25 year old pipe fitter, explained that when he went to clubs and took women home, the result was being irresponsible and having unprotected sex:

"*I've been drinking, just being in the room... the heat of the moment ... the heat of passion, just the heat of the moment taking away all the common sense.*"

Clarence is living with the consequences of having sex with strangers in a mood altered state. Although he did not contract HIV, he expressed that alcohol and unprotected sex resulted in him contracting hepatitis A.:

"*Alcohol did that to me. I had sex with many women that I didn't know had the disease or nothing and I caught hepatitis A behind it once.*"

Like Austin and Clarence, Jason attributed his involvement in HIV risk situations as resulting from substance abuse. He stated:

"*You don't mean to do it, but any mind-altering drug will lead you into a situation where you will have unprotected sex.*"

Joe, a 24 year old truck driver explained that he has unprotected sex in the heat of the moment and once he becomes involved in a sexual act, contact will occur without condom use.

"*Once you get in a certain mood and you get stuck, I'm in that freak mode; there ain't no protection.*"

Harry, a 23 year old landscaper, described how he was told that his partner has AIDS but he continues to have sex with her but gets himself tested for HIV every 6 months. He, in effect, continues to take a chance and uses the test as a way to keep himself informed.

"*In fact, I had two girls and they telling me that you know your partner tell you where they got AIDS from her, but yet and still you get yourself checked and you don't come up with AIDS. You know there's a doubt there somewhere. Every six months I have myself checked for almost a year because I supposed to get it from her. How did I miss it? I don't know.*"

### Barriers to practicing safe sex

Like their perceptions about the reasons for the rapid spread of HIV and the situations that have placed them at risk for infection, one-third of men stated that substance abuse is a barrier to safe sex practices. Other reasons stated as barriers were lack of money for condoms and judging a partner on appearance alone. About one-fifth of participants said they had no barriers to practicing safe sex.

Karl believes that alcohol and drug use, specifically crack cocaine, creates a feeling of power over women which results in the women failing to negotiate safe sex practices. He said:

"*I think alcohol and drugs, mood altering, mind altering, especially with that crack cocaine. When you get that crack cocaine, the fist thing you get, and you take you a hit, you automatically assume power. You know with that crack, you have power over women. You know you can make them do what you want them to do.*"

Freddy, a 28-year old laborer, explained that when a choice between drugs and condom has to be made, he will choose drugs. He stated that he did not have money to buy both condoms and drugs:

"*I don't have enough money to go out and buy condoms that everybody wants. I need to spend money on condoms or I'm a spend it on getting the next hit.*"

Although infrequently stated, Jason said that he had, in the past, judged a woman's HIV/AIDS status based on her appearance:

"*What prevented me from practicing safe sex in the past was, like I said, the way she looked. You feel that you know her, but now I see that you can't go on that.*"

Barry, a 26-year old construction worker, stated that he always uses protection. He said:

"*Like I said earlier, if I can't find a plastic baggie or the plastic that you break on in, I wear a sandwich bag, hey, if I can't use that, I won't mess around. I won't do it!*"

### Facilitators to safe sex practices

Close to one-half of men were motivated to practice safe sex because they were personally acquainted with someone who had been affected by AIDS and had seen its effects firsthand. A quarter of men was adamant about remaining disease free or had previously acquired sexually transmitted infections. About 15% simply stated that life was their motivation.

Barry has seen the effects of AIDS firsthand and the images have caused him to use condoms. He said:

"*I was about to do it with no condom and that seriously woke me up, put a stop and then God stepped in and then about a year later, she passed. The loss of friends and hearing people over the news. Their body just shrivel up cause the body can't hold their bladder, their bowels I mean it's the ugliest sight to see. You know and that's enough to wake you up.*"

Austin, who in the past had taken strangers home and engaged in unprotected sex, explains that his concern for his future facilitates his use of safe sex practices:

"*My motivation is AIDS, not contracting AIDS. The fact that I don't have any kids or I'm not married ... something that will affect my future. I mean I want kids. I wanna be married. I mean if I have AIDS or what have you, don't none of that happen.*"

Jason reiterates Austin's comments quite simply when he states:

"*I want to live. That's the bottom line.*"

Motivation for safe sex practices was not always associated with action even in the face of prior sexually transmitted infection.

Joe adds:

"*One thing that motivates me is, I don't necessarily take heed to it though, but the only venereal disease that I have had in the past is them crabs or whatever you want to call it. One thing that motivates me is that I had got crabs and I felt dirty, I mean I felt real bad.*"

### Strategies for preventing HIV infection

In light of their experiences, participants were asked to describe what they would recommend to curtail the spread of AIDS in the African-American community including what specific governmental, media and community interventions they would consider effective. A variety of recommendations were offered, but the majority believed that more education is needed. Other men suggested that condoms are distributed without charge and consistent condom use is urged. To recruit African Americans into HIV training programs, financial and social incentives were highly recommended as were the use of a community-based, community-friendly approach to program recruitment and implementation.

Trevor, a 27-year old who is unemployed said:

'*The way that AIDS can be prevented is just through education, you know, education and that's basically the only tool that we have is education. Uh, I think the parents play a good role in it [education] too.*"

Wally, a 20-year old bricklayer adds:

"*There's only one way and it's through education and I mean education will sum it up. We have to educate on drugs, we have to educate on protective sex. I mean, education is it.*"

Freddy echoes the sentiments of Trevor and Wally regarding education but also discussed the need to provide condoms:

"*We need to get more condoms out on the streets. All STDs have to be prevented so we have to get more education on the streets, not just about AIDS because they [AIDS and other STDs] work hand in hand.*"

Incentives such as gifts and social events were recommended as a means to get African-American participation in HIV prevention and risk reduction interventions. Wally stated the need to provide incentives to increase program participation:

"*You can give and receive at the same time. What I'm saying is you may have to start having a little gift or something to get them to come in the first few times, or a meal.*"

Barry adds:

"*You gotta give something to get something. Another thing, have you a little barbecue, have it sitting out or something and you can get a whole lot of people. And believe it or not, a crowd of people will open up to a conversation, too.*"

Karl stressed incentives and programs for adolescents:

"*Sometimes especially with the younger generation, you have to give them something that they want. Give them caps, give them T-shirts, but at the same time, push the condoms.*"

Outreach workers were advised to eliminate formality and go into the community to provide their programs. They should make an effort to ensure the target community members are comfortable with their presence. Jason said:

"*And go into the neighborhoods and walk around and talk to folks. People have a tendency to stay away from people in a suit and tie in a neighborhood.*"

Gene, a 20-year old laborer added:

"*Instead of making it formal, just come in off the streets. Go to the people and give them that incentive and sit down and talk to them.*"

It is believed among two-thirds of men participating in these discussion groups that the government can assist AIDS prevention by firstly, providing funding for community-based education programs, secondly, providing convenient and free testing, and thirdly, free condom distribution. Other suggestions included quarantining or having a salient means of identifying those affected by HIV and AIDS, providing more medication and treatment for those affected, and having stiffer penalties for those who knowingly transmit the virus.

Jason believes in the efficacy of outreach programs that provide free testing and incentives:

"*Just have three of four vans, just go to different neighborhood, have free testing, give T-shirts.*"

Terry believes in the community approach to governmental intervention:

"*I think the greatest weapon we have against AIDS is knowledge and we must bring some type of community-based program.*"

Roger asserted his belief that the entire US population should be tested and those with HIV and AIDS identified and placed in isolation:

"*Either quarantine, mandatory testing for HIV for the whole US population. You go to get tested. You're going to a quarantine island for the rest of your life.*"

Terry believes that the government should impose stringent punishment against persons who knowingly infect others with HIV:

"*I think we can make stiffer penalties for people with AIDS that are willingly passing the disease on. Probably the government can open some more clinics or help the doctors find a cure.*"

To create a video that addresses HIV prevention, close to one-half of participants recommended using a sensational approach that includes those morbidly affected by HIV and AIDS. They believe that the video should include footage of people in pain, being shunned by friends and family, and suffering tremendous physical pain. They also recommended testimonials from infected persons. A quarter of respondents believed that the video should be educational providing information about transmission and prevention. The remainder suggested the use of rappers to promote the message of prevention and acknowledgement that HIV happens to African Americans.

Karl who has a college degree stated that a video developed for AIDS prevention should include the disease's effects on the person:

"*They need to see those people suffering to let them know that this is how you're going to end up if you don't begin to practice safe sex. But also how people are being discriminatory to you, treating you like crap like you're this or that or you're contagious or something.*"

Roger has similar views but adds that education about high risk behaviors should be included:

"*Stay away from the dope and don't go tricking and also go to the hospitals. A lot of African Americans are not educated on the full blown AIDS and how it destroys the major organs in your body. And let them see first hand on what you're dealing with here.*"

Jason would like a video that includes the various modes of transmission as well as prevention information:

"*Just about everything that you can get on it from drug use to safe sex to having sex with the same partner. You know it's different ways you can catch it and a lot of people don't know that.*"

When asked what can be done to recruit African Americans to HIV training programs, most men suggested the use of participation incentives, especially those that are financial and preventive (condoms). The men also recommended recruiting participants from and having the programs take place within the communities in which the target population resides.

Wally said:

"*You may have to start with a few passes; they can be little small things. Then everyone would gradually grab a hold and then their minds would be off on what you're giving personally and they could see what they can receive. You can give and receive at the same time. What I'm saying is you may have to start having a little gift or something to get them to come in the first few times, or a meal.*"

Jason suggests community-based recruitment:

"*Stand at every corner. AIDS is gonna get too bad in a couple of years. We don't want it to be too late before we say, "Hey, let's put education groups here and people just stop here and get rubbers.*"

## Conclusions

The results indicate that the respondents taking part in this study believe that HIV and AIDS are continued threats to the African-American community because of sexual risk taking behavior, that is, failure to use condoms. Further, African-American men are having sex without condoms when having sex with women often when they are under the influence of alcohol or other mind-altering substances and they are having sex with men while incarcerated and become infected and once released resume unprotected sexual relations with women. According to the men, substance abuse is an important part of the problem of HIV in the African-American community. This is in keeping with research that shows that drug use, especially crack cocaine, is linked to sexual risk taking among African Americans and to increased likelihood of becoming infected with other sexually transmitted diseases (STDs) including HIV [27,28]. African Americans are disproportionately affected by STDs [1] and African-American crack users are more likely to have multiple partners and to participate in drug for sex exchanges [29,30]. According to the men interviewed, they were less likely to use a condom when under the influence. Research shows that crack cocaine users may not perceive sexual risk taking as an important self-threat compared to other social and health issues they confront on a daily basis [27].

Although most respondents indicated they have knowledge of behaviors that place one at risk for HIV and they are motivated by reasons such as life and not wanting to contract an STD, they still fail to consistently use condoms and may even have sex with someone they know is HIV positive and they continue to suggest that more educational programs are needed. This shows that knowledge and motivation are not enough for behavior change to occur. These men need education but education that is provided in a way that has been different from what has been offered in the past because it seems not to change their behaviors. Interventions for men should address condom use, condom availability, skills for using condoms, and eroticizing condoms. Substance use programs should incorporate sexual risks. Men in the present study strongly recommended that HIV/AIDS videotaped messages should include footage of the sensational effects of the disease. There is a trend toward reality broadcasting in the US and development and field testing of such a videotape for AIDS prevention might prove worthwhile [31]. Low-income women taking part in a similar focused group discussion also recommended the use of sensationalism. Contrary to research indicating fear or sensationalism does not work [32-34], based on these results it may be worthwhile to field test such a videotape with groups of African Americans to evaluate the utility of such a teaching tool. If the tide of HIV and AIDS infection among African Americans is to be reduced, programs must incorporate culturally relevant contextual information presented to the target audience in a setting and in a manner that addresses their norms and beliefs and provides them the knowledge and skills needed to make correct decisions. The message could be presented using behavioral journalism, an approach espoused by McAlister [35] that offers a balance between the message source and the audience constructing messages within a theoretic framework and tailoring them to specific audiences. It may well be that this approach works with low-income African Americans who are engaged in high risk behaviors. Such a program could also include information about transmission routes, a subject identified as important to discuss when developing training programs.

Prior research has identified the leading causes of HIV infection among African-American men that includes sexual contact between African-American men who have sex with men, injection drug use and heterosexual contact. These high-risk behaviors are exacerbated by the high rate of poverty among African Americans that may be due to high rates of unemployment and associated drug related economic activities, injection drug, and substance abusers' engagement in unprotected sex when they are under the influence of drugs and alcohol [1,13]. The present study sought to identify the sociocultural contexts of sexual risk taking among African-American men and to gather information that could be used to develop and disseminate a videotape-based HIV prevention intervention for African-American men. Although the CDC identifies the leading cause of HIV transmission among African-American men as homosexual contact, none of the participants in the present study acknowledged this cause [1]. Men of color who have sex with other men often do so without disclosure and may consider themselves heterosexual [36]. Further research is needed in this area to determine how best to address this issue within the cultural context of African Americans and non-disclosing men. Lack of money for condoms was identified as a barrier to condom use. Many of the men believed that condoms should be a part of any HIV training program and that they could also be given as participation incentives for coming to a training program. Prior research has indicated that a combined approach to HIV prevention that includes HIV testing and counseling, educational and behavioral interventions delivered through community outreach, condom distribution and substance abuse treatment are effective means for reducing transmission [37]. However, such interventions have not yet been widely implemented in a sustained and integrated fashion.

Although most of the men had completed high school or beyond, they were mainly homeless or living in substandard housing. Their conditions of poverty may exacerbate their risk for infection because they may engage in illegal or high-risk behaviors. The barriers these men face related to poverty should also be addressed when developing training programs. This may call for the involvement of social service workers. Like programs developed for African-American women, they must be presented in settings that are familiar, comfortable and easily accessible to the target audience and incorporate culturally relevant contextual information [38].

Before developing programs, health educators should become familiar with the facilitators and barriers to HIV risk reduction behaviors among this population. They should also recognize that African Americans are a heterogeneous group and that not all messages will work with all audiences. The messages must be tailored and celebrate the diversity that exists within this population. Interventions such as videotapes should have mass appeal yet contain contextual, cultural, and gender specific messages.

There are several limitations to this study. Qualitative data collection methodology was employed and it is therefore not possible to make assumptions or draw inferences. Next, the data cannot be generalized to other African American men. The participants were homeless and low-income and selected using a convenience sampling approach. The goal of this study, however, was to recruit low-income African-American men because by virtue of their income status they may be at increased risk for HIV infection. Although the findings from this research are limited, they may provide a foundation for conducting future research among and developing a videotape-based HIV intervention for low-income African-American men.

## Competing Interests

The author(s) declare that they have no competing interests.

## Authors' contributions

EJE, AFM and GOO conceived and designed the study. EJE, AFM, RJP jointly planned and executed the data analyses. EJE and AFM wrote the paper with assistance from RJP, GOO and NIO.

**Table 1:** Focus group guide questions

1. Do you perceive AIDS as a threat to the African American community and why?
2. What are the perceived roles of men in heterosexual relationships in the African American community?
3. What are the expectations for personal and sexual responsibilities for contraception and sexually transmitted diseases prevention among African American men?
4. What situations have placed you at risk for HIV infection in the past?
5 How have alcohol and drug use placed you at risk of HIV infection?
6. What are the things that motivate you to practice safe sex?
7. What are the things or barriers that prevent you from practicing safe sex
8. Why do you think that AIDS is spreading so rapidly in the African American community?
9. What information do you think we need to include in a videotape developed to train African Americans about HIV prevention that will encourage them to watch the videotapes?
10. Do you have any other suggestions on how AIDS can be prevented in the African American community?
11. What can we do to get people to sign up for focus groups such as this one and also get them to participate in HIV/AIDS training programs?
12. What can we do to make these training programs most useful to you?

## Pre-publication history

The pre-publication history for this paper can be accessed here:


